# Phylogenetic relationship and characterization of the complete chloroplast genome of the alpine leaf-warbler in Qinghai-Tibet Plateau

**DOI:** 10.1080/23802359.2019.1677184

**Published:** 2019-10-15

**Authors:** Zhenhao Liu, Jinlong Liu, Xiangwu Chen, Zhenyu Jin, An Luo, Shaobin Li

**Affiliations:** a College of Life Science, Yangtze University, Jingzhou, China;; b College of Agriculture, Yangtze University, Jingzhou, China

**Keywords:** Bush habitat, mitochondrial genome, passerine, *Phylloscopus affinis*, Phylogeny, West Chinese leaf-warbler

## Abstract

The Alpine Leaf-warbler (*Phylloscopus occisinensis*) is a small-sized and poorly-known songbird endemic to China. In this study, we sequenced and described the whole mitochondrial genome of the Alpine leaf-warbler. The entire mitochondrial sequence was determined by long-range PCR and conserved primer walking approaches. The results demonstrated that the whole mitochondrial genome of *P. occisinensis* was 16,879 bp in length with 53.1% A + T content; the genome harboured the same gene order as that of other passerine birds, including 13 protein-coding genes, 2 rRNA genes, 22 tRNA genes and non-coding control region. The control region (D-loop) of *P. rubicilloides* include two portions that one was located between tRNA-Gln and tRNA-Phe (219 bp in length) and the other is between tRNA-Thr and tRNA- Pro (1087 bp in length), which is similar to other Leaf-warblers. Phylogenetic analysis indicated that the genome of *Phylloscopus* species clustered within a clade and is closer related to Aegithalidae species than Paridae species. These mitochondrial data are potentially important for the further studies on molecular evolution and conservation genetics on leaf warbler species.

More than 60 leaf-warbler species were included in genera *Phylloscopus* (Clements et al. 2018; Gill and Donsker 2019), and most of these genera are poorly known on their natural history (Xiao et al. 2017; Fjeldså 2019). The alpine leaf-warbler *P. occisinensis* is a middle-sized member of *Phylloscopus*. The birds are endemic to China and occur in the eastern Qinghai-Tibet plateau, Sichuan and Gansu province (Martens and Trautmann 2008; Clements et al. 2018). It was generally considered to be a subspecies of Tickell’s Leaf Warbler *P. affinis*, but now it is split from *P. affinis* (Martens et al. 2008). The breeding habitat of this species is typically rocky and bushy habitat in dry barren mountains and alpine valley, and their altitudinal distribution ranges from 1000 m to even more than 4000 m altitude (Lu 2008; Martens et al. 2008; Fjeldså 2019). Currently, the Alpine leaf-warbler is still poorly understood and the knowledge on its natural history is mainly based on simple descriptions from species accounts (Martens and Trautmann 2008; Clements et al. 2018), and only a few mitochondrial genes are available on this species in genbank database. In this study, we amplified sequenced the whole mitochondrial genome of *P. occisinensis* by La-PCR. The newly sequenced complete mitochondrial genome sequence will provide basic data for further evolutionary studies on these genera.

An embryo (id alw_001) of this species was sampled in an unhatched egg from an abandoned nest, on June 21th 2017 at bush habitat of Tianjun County (37°18′N, 99° 01′E; 3416 m altitude), northeastern of Tibet Plateau (details of the habitat are available in Li et al. 2018). The sample was immediately stored in alcohol. Genomic DNA was extracted from partial of the sample according to the protocol of TIANamp Genomic DNA kits (Tiangen, Beijing). The remaining sample is now stored in –20 °C cold refrigerator in specimen room 1-317, No. 1 Teaching Building of Yangtze University (No. 88 Jingmi Road, Jingzhou district, Jingzhou, China, postcode 434025). The complete sequence of the Alpine leaf-warbler mitochondrial genome was determined by long-range PCR and conserved primer walking approaches.

The results revealed that the entire mitochondrial genome of the Alpine leaf-warbler comprised 16,879 bp nucleotides in length, which contained the typical mitochondrial structure of passerine birds (Cao et al. 2017; Jiao et al. 2018), including 13 protein-coding genes, 22 tRNA genes, 2 rRNA genes and non-coding control region. The control region of *P. occisinensis* has two portions, which is similar to two other leaf warbler species *P. proregulus* and *P. inornatus* (KF742677, Jiao et al. 2018). One portion of the control region is 219 bp length between tRNA-Gln and tRNA-Phe and the other is 1087 bp in length between tRNA-Thr and tRNA-Pro, which contained several conserved sequences involved in the replication and transcription of mitochondrial genome. The overall nucleotide composition includes A (29.79%), T (23.31%), G (14.58%) and C (32.32%), with a total A and T content of 53.10%.

Among the 37 mitochondria genes in *P. occisinensis*, 28 genes were encoded on heavy strand and the remaining genes on the light strand. All protein-coding genes of the *P. occisinensis* mitochondrial genome started with ATG codon, except for ND3 with ATA. For terminate codon usage, most of the genes terminate with TAA or TAG, COX1 terminated with AGG, ND5 terminated with AGA and the COX3 and ND4 genes had an incomplete termination codon T. The whole genome sequence has been deposited in GenBank with the accession number of MK513447.

Phylogenetic analyses were executed with complete mitochondrial genomes of this study and 27 other related bird species from the GenBank database. The topology of the phynogeny was inferred using Neighbor-Joining analyses in the programme MEGA7 (Kumar et al. 2016). Models on almost all nodes were statistically well supported by a high bootstrap values at most nodes (Figure 1). The phylogeny revealed that three *Phylloscopus* species clustered in a clad and were closer related to Aegithalidae species than Paridae. All the clades were consistent with the traditional morphology-based taxonomies and molecular taxonomies (Clements et al. 2018; Gill and Donsker 2019). Up to date, most of the *Phylloscopus* species are still poorly known on both their natural history and genetic information (Xiao et al. 2017; Gill and Donsker 2019), and only two complete mitochondrial genome of *Phylloscopus* species have released in Genebank (Figure 1). The additional complete mitochondrial genome of the Alpine leaf-warbler could provide fundamental data for further genetic conservation and phylogenetic studies on *Phylloscopus* species.

**Figure 1.**
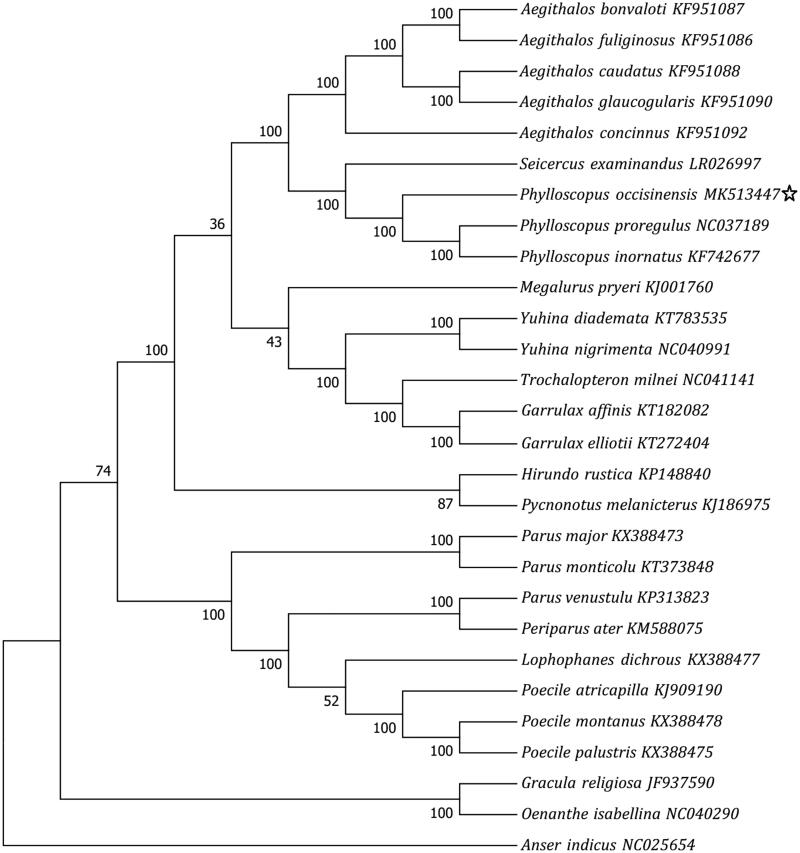
Phylogenetic relationships of 28 taxa were inferred with the neighbor-joining method based on their whole mitochondrial genome (the scientific name followed by its genbank accession number; data of this study were marked with a star; numbers at branches reveal bootstrap values from 1000 replications.
